# Reducing intrusive traumatic memories after emergency caesarean section: A proof-of-principle randomized controlled study

**DOI:** 10.1016/j.brat.2017.03.018

**Published:** 2017-07

**Authors:** Antje Horsch, Yvan Vial, Céline Favrod, Mathilde Morisod Harari, Simon E. Blackwell, Peter Watson, Lalitha Iyadurai, Michael B. Bonsall, Emily A. Holmes

**Affiliations:** a Department Woman-Mother-Child, University Hospital Lausanne, Lausanne, Switzerland; b Department of Endocrinology, Diabetes, and Metabolism, University Hospital Lausanne, Lausanne, Switzerland; c Department of Child and Adolescent Psychiatry, University Hospital Lausanne, Lausanne, Switzerland; d Mental Health Research and Treatment Center, Ruhr-Universität Bochum, Bochum, Germany; e Medical Research Council Cognition and Brain Sciences Unit, Cambridge, UK; f Department of Psychiatry, University of Oxford, Oxford, UK; g Department of Zoology, University of Oxford, Oxford, UK; h Department of Clinical Neuroscience, Karolinska Institutet, Stockholm, Sweden

**Keywords:** Posttraumatic stress disorder, Acute stress disorder, Early intervention, Proof-of-principle randomized controlled study, Cognitive, Computerized, Childbirth, Universal intervention, ECS, emergency caesarean section, PTSD, posttraumatic stress disorder, ASD, acute stress disorder, ASDS, Acute Stress Disorder Scale, HADS, Hospital Anxiety and Depression Scale, PDS, Posttraumatic Diagnostic Scale

## Abstract

Preventative psychological interventions to aid women after traumatic childbirth are needed. This proof-of-principle randomized controlled study evaluated whether the number of intrusive traumatic memories mothers experience after emergency caesarean section (ECS) could be reduced by a brief cognitive intervention. 56 women after ECS were randomized to one of two parallel groups in a 1:1 ratio: intervention (usual care plus cognitive task procedure) or control (usual care). The intervention group engaged in a visuospatial task (computer-game ‘Tetris’ via a handheld gaming device) for 15 min within six hours following their ECS. The primary outcome was the number of intrusive traumatic memories related to the ECS recorded in a diary for the week post-ECS. As predicted, compared with controls, the intervention group reported fewer intrusive traumatic memories (*M* = 4.77, *SD* = 10.71 vs. *M* = 9.22, *SD* = 10.69, *d* = 0.647 [95% CI: 0.106, 1.182]) over 1 week (intention-to-treat analyses, primary outcome). There was a trend towards reduced acute stress re-experiencing symptoms (*d* = 0.503 [95% CI: −0.032, 1.033]) after 1 week (intention-to-treat analyses). Times series analysis on daily intrusions data confirmed the predicted difference between groups. 72% of women rated the intervention “rather” to “extremely” acceptable. This represents a first step in the development of an early (and potentially universal) intervention to prevent postnatal posttraumatic stress symptoms that may benefit both mother and child.

**Clinical trial registration:**

ClinicalTrials.gov, www.clinicaltrials.gov, NCT02502513.

## Highlights

• An intervention to reduce traumatic memories in mothers after ECS was tested.
• We compared usual care plus visuospatial cognitive task with usual care.
• After 1 week, the intervention group reported fewer intrusive traumatic memories.
• They also showed a trend towards reduced acute stress re-experiencing symptoms after 1 week.
• This early game-based computerized intervention may benefit both mother and child.

Operative delivery by emergency caesarean section (ECS) is indicated in cases of risk to maternal and/or fetal life, therefore qualifying as a psychologically traumatic event for the mother (American Psychiatric Association, 2013). Even when the baby is delivered safely, by one month post-ECS approximately 39% of mothers have developed postnatal posttraumatic stress disorder (PTSD) (Soderquist, Wijma, & Wijma, 2002). Posttraumatic stress disorder consists of four symptom clusters: re-experiencing (including intrusive traumatic memories of the event), avoidance, hyperarousal, and negative cognitions and mood (American Psychiatric Association, 2013).

Recurrent and distressing traumatic intrusive memories consist of involuntary, sensory (predominantly visual) mental images which intrude the mind unbidden, their content often overlapping with the most distressing moments of the traumatic event (American Psychiatric Association, 2013, Grey et al., 2001). After ECS, examples of traumatic intrusive memories include a mental image springing to mind of the screen of the fetal heart rate monitor indicating ‘Stop’ or seeing the face of the doctor announcing that the patient immediately needs an ECS.

Intrusive traumatic memories are the core clinical feature of both acute stress disorder (ASD) and PTSD (American Psychiatric Association, 2013, Brewin and Holmes, 2003). As a precursor of PTSD (which is diagnosable from 1-month post-trauma), women may experience ASD symptoms such as intrusive traumatic memories in the first four weeks after ECS (Harvey & Bryant, 2000). Indeed, traumatic intrusions and other ASD symptoms in the first 10 days in a sample of patients presenting to a hospital emergency department following motor vehicle accidents, terrorist attacks, or work accidents have been found to predict chronic PTSD (Galatzer-Levy, Karstoft, Statnikov, & Shalev, 2014). Thus, reducing intrusions in the acute period may be beneficial not only in its own right for reducing distress, but for reducing later PTSD. While the prevalence rate of ASD following ECS is unknown, it is likely to be substantially higher than that after childbirth generally (5.6%) (Creedy, Shochet, & Horsfall, 2000). Critically, targeting early symptoms is useful in its own right for mother and child, and may ultimately help prevent later PTSD (McKenzie-McHarg et al., 2015).

Early symptoms soon after childbirth (such as being “haunted by” intrusive images of the traumatic birth (Fenech & Thomson, 2014)) are highly distressing for women. Traumatic intrusive images may be associated with sleep problems and dysfunctional coping mechanisms, such as non-initiation or early cessation of breastfeeding in order to avoid those images often triggered by close contact with their baby (Beck and Watson, 2008, Fenech and Thomson, 2014). There is mounting evidence that later postnatal PTSD symptoms can negatively affect the attachment relationship between the baby and the mother, increase parenting stress, and compromise the baby's subsequent development (Fenech and Thomson, 2014, McDonald et al., 2011, Parfitt and Ayers, 2009, Parfitt et al., 2014). Postnatal PTSD also negatively influences future reproductive choices, can lead to fear of childbirth (tokophobia), sexual problems, avoidance of medical care (King et al., 2017, Morland et al., 2007), and increases the risk of maternal stress and negative birth outcomes during a subsequent pregnancy (Seng et al., 2001). Postnatal PTSD significantly contributes to the costs of perinatal mental health problems, estimated at £8.1 billion per year in the UK alone (Bauer, Parsonage, Knapp, Iemmi, & Adelaja, 2014).

Interventions are urgently needed to prevent the development of postnatal post-traumatic stress and acute posttraumatic stress reactions as their precursor. Given in ECS that there is both a known traumatic cause, and a substantial rate of subsequent mental health impairment, it is critical both for mother and child that interventions are developed. However, to date, we lack evidence-based interventions for women after traumatic childbirth (Bastos, Furuta, Small, McKenzie-McHarg, & Bick, 2015), particularly those targeting early symptoms of posttraumatic stress that could improve longer term outcomes (McKenzie-McHarg et al., 2015). Here we investigate a new preventative intervention to reduce intrusive memories of the traumatic event, taking an innovative hypothesis-driven approach (Holmes, Craske, & Graybiel, 2014) informed by cognitive science of emotional memory and using technology (computer game play) rather than a therapist.

Our hypothesis is to reduce the frequency of recurrence of traumatic memories (e.g. the upsetting intrusive visual memories of the heart rate monitor/doctor's face in the patient examples given earlier) via a “cognitive therapeutic vaccine” informed by cognitive science (Holmes et al., 2010, Poland et al., 2002). This approach is informed by a number of insights: First, intrusive memories of trauma comprise sensory-perceptual mental images with visuospatial components (Brewin, 2014, Holmes et al., 2005). They are proposed to occur due to excessive perceptual (sensory) processing during a trauma (Brewin and Holmes, 2003, Brewin, 2014, Ehlers and Clark, 2000, Holmes and Bourne, 2008) resulting in sensory-based (predominantly visual) images of the trauma that intrude into the mind spontaneously. Second, cognitive psychology research suggests that we can disrupt visual aspects of (traumatic) memory that underpin intrusions by actively engaging in visuospatial tasks, since these compete for resources with the brain's sensory-perceptual resources (Andrade et al., 1997, Baddeley and Andrade, 2000, Kavanagh et al., 2001). Numerous types of visuospatial tasks could be used – here we use Tetris game-play in translating laboratory work to the clinic.

Third, neuroscience research on the formation of memory and its consolidation suggests that memories are malleable (i.e. not yet stabilised) from the onset event until approximately 6 h after initial encoding (McGaugh, 2000, Nader et al., 2000, Walker et al., 2003). This early time frame post-trauma presents a window of opportunity during which to disrupt the consolidation of visual (emotional) memory – here with a visuospatial task. Selectively disrupting the visual aspects of trauma memory during its consolidation is predicted to render the memory less ‘overly perceptual’ and thus less intrusive.

Fourth, our cognitive behavioural formulation about trauma memory suggests that it is only discrete points within the memory (and not others) – i.e. ‘hotspots’ – that later become intrusive memories (Grey and Holmes, 2008, Grey et al., 2002, Holmes et al., 2005) (see also (Bourne et al., 2013, Clark et al., 2014)). Thus we suggest that one does not need to engage in the competing task for the full duration of the original trauma, but rather adequately compete for resources with the consolidation of these (briefer) hotspot moments selectively, intrusions of which patients typically begin to re-experience even soon after the event. Laboratory studies indicate that participants should engage in the competing task uninterrupted for approximately 10–20 min (Holmes et al., 2009, Holmes et al., 2010, James et al., 2015). Given that the women were still receiving care in the hospital at the time of the intervention, the hospital context provided an in vivo cue for the trauma memory hotspots of the ECS.

In summary, actively engaging in a visuospatial cognitive task for c.15 min up to 6 h after a traumatic event is predicted to reduce the occurrence of subsequent intrusive memories of the trauma via competing with sensory aspects of the trauma memory before it has been fully consolidated. Indeed, lab-based experiments with healthy volunteers have demonstrated that engaging in the computer game Tetris, a game which taxes visuospatial functions (Green & Bavelier, 2003), up to 4 h following exposure to traumatic film material can significantly reduce the later number of intrusive memories (Holmes et al., 2009, Holmes et al., 2010). The requirement for the task to compete specifically for visuospatial resources (as opposed to merely providing distraction) has been indicated by findings that non-visuospatial tasks (e.g. a verbal computer game) do not reduce, and in some instances may even increase, the occurrence of intrusive memories (Holmes et al., 2010). We note that in addition to Tetris, other absorbing visuospatial tasks would also be predicted to be beneficial.

Women hospitalised following an ECS are an ideal population for testing the potential clinical application of this cognitive science paradigm in reducing intrusive memories of their traumatic experience, due to the following features: (1) a homogenous, single-event adult trauma, (2) a relatively homogeneous population in a tightly controlled environment, (3) the intervention takes place in the context where the trauma occurred i.e. on the hospital ward itself (4) the possibility to deploy technological interventions e.g. via a handheld gaming device (5) the great need for preventative interventions against post-traumatic stress for mothers and babies. This translational study aimed to test the primary hypothesis that post-ECS, mothers who perform a visuospatial cognitive task (Tetris computer-game play) for 15 min via Nintendo DS within the first 6 h following their ECS while in the hospital alongside usual care will have fewer intrusive memories at 1 week compared with the control group (usual care only). Secondary hypotheses were: (1) the Tetris invention group compared with the control group would have less acute traumatic stress symptoms at 1 week, and posttraumatic stress symptoms at 1 month; (2) playing Tetris will be rated as an acceptable intervention by patients.

## 1 Materials and methods

**Fig. 1:**
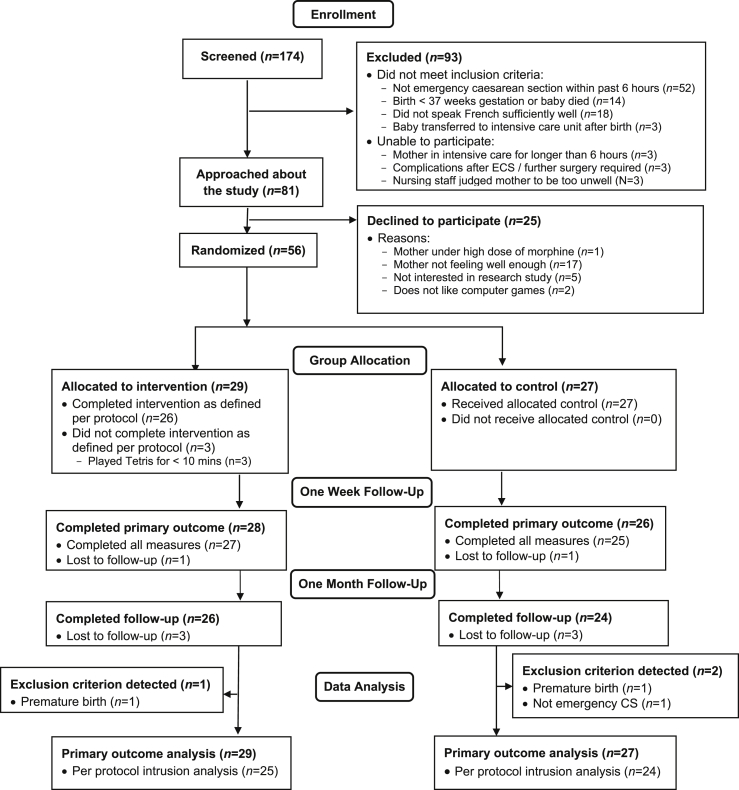
Study flowchart.

Women (over 18 years) who had undergone an emergency caesarean section (ECS) and given birth to a live baby at term in the previous 6 h in the maternity department of a Swiss University hospital were included. Women were screened for the following exclusion criteria: (a) planned caesarean section, (b) insufficient French language skills, and (c) baby transferred to neonatal intensive care unit (required by the ethics committee to avoid excessive emotional burden for the participants). Screening took place whilst mothers were in the wake-up room of the maternity hospital, usually with their baby at their side. Those who were eligible were informed about the study and asked for their written informed consent. Of 81 women eligible to participate, 25 declined participation and 56 women were enrolled. For three women, it was subsequently detected (e.g. on receiving obstetric data at the end of the study) that full inclusion criteria had not been met (e.g. birth was premature, Fig. 1). Table 1 presents baseline data for the sample. Participants had a mean age of 33.39 (4.22) years, with a Swiss or other European background. The majority of women had a partner and a university degree.

**Table 1:** Demographic characteristics, obstetric and neonatal variables, and mental health symptoms at baseline for intervention and control groups.

	Intervention (*n* = 29)	Control (*n* = 27)	*p*
***Demographic variables***			
Age (years; mean, SD)	34.24 ± 3.81	32.37 ± 4.47	0.096
Nationality (N, %)			0.257
Swiss	13 (45%)	13 (48%)	
Other European	15 (52%)	10 (37%)	
Non-European	1 (3%)	4 (15%)	
Marital status (N,%)			0.671
Single	4 (14%)	2 (7%)	
Married/Co-habiting	25 (86%)	24 (89%)	
Divorced	0 (0%)	1 (4%)	
Education (N,%)			0.752
Primary school	1 (3%)	1 (4%)	
Middle school	3 (10%)	4 (15%)	
Secondary/high school	1 (3%)	3 (11%)	
Apprenticeship	3 (10%)	4 (15%)	
University	21 (72%)	15 (56%)	
Largo score (mean, SD)	2.95 ± 0.90	2.76 ± 0.87	0.438
***Obstetric variables***			
Duration of labour (min; mean, SD)	243.20 ± 258.22	264.93 ± 221.60	0.746
Dilation at time of ECS (cm; mean, SD)	5.08 ± 4.74	5.96 ± 4.40	0.484
Cord blood 1 pH (mean, SD)	7.23 ± 0.06	7.26 ± 0.13	0.474
Cord blood 2 pH (mean, SD)	7.31 ± 0.06	7.26 ± 0.03	0.072
Amount of blood loss (ml; mean, SD)	515.52 ± 253.93	529.63 ± 185.67	0.814
Parity (mean, SD)	1.45 ± 0.69	1.48 ± 0.75	0.864
***Neonatal variables***			
Gestational age (weeks; mean, SD)	39.41 ± 1.55	39.48 ± 1.89	0.884
Birth weight (g; mean, SD)	3269.66 ± 578.99	3373.33 ± 496.07	0.476
Apgar score 1 (mean, SD)	7.24 ± 2.92	7.81 ± 2.51	0.447
Apgar score 2 (mean, SD)	9.28 ± 1.0	9.04 ± 1.25	0.437
Apgar score 3 (mean, SD)	9.62 ± 0.56	9.54 ± 0.9	0.702
***Mental health symptoms***			
ASDS re-experiencing subscale (mean, SD)	5.69 ± 1.87	6.37 ± 3.27	0.900
ASDS avoidance subscale (mean, SD)	2.14 ± 0.44	2.07 ± 0.27	0.678
ASDS dissociation subscale (mean, SD)	4.48 ± 1.81	4.74 ± 1.91	0.535
ASDS arousal subscale (mean, SD)	7.00 ± 2.27	8.37 ± 4.20	0.354
ASDS Total score (mean, SD)	19.32 ± 4.58	21.58 ± 8.06	0.495
HADS Anxiety (mean, SD)	5.76 ± 3.04	6.07 ± 3.76	0.731
HADS Depression (mean, SD)	2.55 ± 1.78	3.04 ± 2.44	0.397

ECS = emergency caesarean section; ASDS = Acute Stress Disorder Scale; HADS = Hospital Anxiety and Depression Scale; PDS = Posttraumatic Diagnostic Scale.

The study was approved by the ethics committee for research in humans of the Canton Vaud, Switzerland (approval number: 480/2012). The study was registered as NCT02502513 (ClinicalTrials.gov) while recruitment was ongoing. Recruitment started in June 2013 and stopped in August 2015 when the target sample size was reached. A planned sample size of *N* = 56 was chosen to have 80% power after possible attrition (10%) to find a between-groups difference of *d* = 0.80 in the primary outcome (see below) at p < 0.05, based on previous laboratory work with healthy participants using an analogue trauma paradigm (Holmes et al., 2009). Holmes et al. (2009) found a large effect size of *d* = 0.91 for the reduction in flashback frequency in the Tetris condition (mean 6.70, SD 5.47) compared to a no-task control condition (mean 2.80, SD 2.65). In the current study, a more conservative effect size of *d* = 0.80 was employed.

The final follow-up data was collected in October 2015. The study initially recruited both mothers and fathers, but recruitment of fathers stopped in February 2014 due to low uptake e.g. they were not present shortly after the birth.

After the written informed consent procedure, participants completed baseline measures including the Hospital Anxiety and Depression Scale (HADS) and sociodemographic questionnaire. The HADS (Zigmond & Snaith, 1983), a 14-item questionnaire, measures state anxiety and depression. Each item is scored from 0 to 3, with higher scores indicating greater symptoms. The Cronbach α for the HADS anxiety subscale for the current study was *α* = 0.734 and for the depression subscale was *α* = 0.612. The sociodemographic questionnaire included questions about participants’ age, marital status, education, and profession. Socio-economic status was calculated based on educational history (1 = primary education, no professional training to 4 = university degree) and current profession (1 = unqualified employment to 4 = managing director or independent academic), with a maximum total score of 4 (Pierrehumbert, Ramstein, Karmaniola, & Halfon, 1996).

To index current distress about the ECS pre-intervention, we used scores from the Acute Stress Disorder Scale (ASDS) (Hansen, Armour, Wang, Elklit, & Bryant, 2015). The ASDS is a 14-item self-report inventory based on DSM-5 criteria (American Psychiatric Association, 2013), here in relation to the ECS trauma experienced in the last 6 h. It consists of three subscales (intrusions, avoidance, negative mood and cognition) and items are rated on a 5-point Likert scale from 1 = *Not at all* to 5 = *Almost always*. The Cronbach α for the current study was *α* = 0.824. Here the baseline ASDS data was used to check that no baseline differences existed between the two groups regarding the experience of distress in the brief period prior to the intervention, but not to measure ASD per se.

Obstetric and neonatal information (see Table 1) was extracted from hospital records by a research assistant blind to group allocation at the end of the study.

After baseline assessment, participants were randomly assigned to one of two parallel groups (intervention vs. control) at a 1:1 ratio using pre-prepared, sealed envelopes. The randomization sequence was generated by a research assistant using a computer-generated simple four-block design and sequentially numbered envelopes were prepared in advance. After conducting the baseline assessment the researcher opened the envelope and announced the group allocation to the participant. Participants in the intervention group were instructed to engage in a cognitive task, the computer game Tetris, for 15 min. Tetris (Version 1.2.1 Blue Planet Software, 2007) requires the player to move and rotate geometrical shapes under time pressure. Seven differently shaped, colored geometric blocks fall from the top to the bottom of the screen in a random sequence one at a time. Using different buttons on a gaming device (Nintendo DS), participants have the choice of moving the blocks left or right, to rotate them 90°, or to accelerate them down as they fall to the bottom of the screen. The aim is to create complete horizontal lines using the blocks, at which point the horizontal line disappears, and the participant is awarded points. Participants were asked to focus on the block due to fall after the one they were currently manipulating, which was shown in a preview in the right upper corner of the screen. To encourage mental rotation (James et al., 2015), participants were instructed to work out in their imagination where best to place each block in order to complete the horizontal lines and advance with the game. Tetris was set to “Marathon” mode with the sound switched off. Participants were required to play Tetris for at least 10 min.

Participants received instructions regarding how to use the Nintendo DS and a trial run of two minutes of game practice. The control group did not play Tetris. Both groups continued receiving routine clinical care. The intervention took place within the first six hours after ECS, whilst participants were still in their hospital bed as determined by medical notes for the time of the ECS. Researchers collaborated closely with the staff in the wake-up room and on the postpartum ward to ensure that the intervention did not interfere with important care procedures, such as first contact with the baby, establishment of breastfeeding, washing of the patient or pain management.

After the intervention/control, both groups kept a daily diary of intrusive traumatic memories related to the ECS for one week, (adapted from Holmes et al., 2009, Holmes et al., 2010, James et al., 2015). The frequency of intrusive traumatic intrusions related to the ECS as reported in the diary was the predefined primary outcome measure. Given the colloquial use of the term “flashback”, this was used to describe intrusive memories, and defined to participants as follows: “Flashbacks are memories of the labor, the emergency caesarean section or childbirth that pop into your mind without warning. They can be vivid and emotional, and are often like visual pictures in your mind's eye e.g. like a snapshot image or a film clip. However, they can involve any senses e.g. sounds, smells and sensations in your body. Flashbacks of the childbirth can be very short, fleeting and broken up. We would still like to know about them in the diary!”.

Participants recorded the occurrence of intrusive traumatic memories in daily life by putting ticks in a box for the relevant day in which the intrusive traumatic memory occurred, or marked “zero” if they experienced no intrusive memories in that day. Additionally, participants were asked to write a brief description for each intrusive memory. Instructions for filling in the diary were provided both orally and in writing in the diary. The diary was started on the day of the caesarean (“Day 1”) and completed for seven days. Participants were asked to keep the diary to hand and make a time each day when they could complete it. Participants and researchers administering the intervention or outcome measures were not blind to participant allocation.

All participants stayed in hospital for at least five days following their ECS. On the third day, a researcher visited the participants on the postpartum ward to remind them about filling in the diary and to answer any questions they might have. At 1 week, all participants were at home and were sent by post the ASDS and HADS (secondary outcomes) and at 1 month the Posttraumatic Diagnostic Scale (PDS; Foa (1995) and HADS for completion (secondary outcomes). The PDS is a 17-item self-report measure (to measure PTSD symptoms). For each item, participants were asked to rate how often they experienced PTSD-symptoms in relation to the ECS in the past month, using a 4-point Likert frequency scale (0 = *Not at all or only one time* to 3 = *5 or more times a week/almost always*). The PDS has three subscales (re-experiencing, numbing/avoidance, hyperarousal). The Cronbach α for the PDS for the current study was *α* = 0.903. Finally, participants in the intervention group were asked to rate how acceptable (1 = *Not at all acceptable* to 5 = *Extremely acceptable*) and how useful (1 = *Not at all useful* to 5 = *Extremely useful*) it had been to play Tetris on a 5-point Likert Scale and whether they would recommend this intervention to a friend (*yes/no*).

### 1.1 Data analysis

Statistical analyses were carried out according to a pre-specified statistical analysis plan by a statistician not involved in recruitment or data collection and blind to group allocation. An interim analysis of group differences in outcomes was conducted in June 2014 after one year of recruitment to secure further internal funding. Analyses were carried out using IBM SPSS version 22. The primary analysis was carried out as intention-to-treat analyses, with missing data imputed via grand mean multiple imputation. Secondary analyses were also conducted in a “per protocol” sample, pre-defined as those participants who i) met all inclusion criteria; ii) played Tetris for at least 10 min (if in the intervention group), and; iii) completed the outcome measure. The aim of the per protocol analyses was to establish the efficacy of the intervention when successfully administered to the target population. The sample sizes for both the intention-to-treat and the “per protocol” analyses are given in Table 2, Table 3. In the group analyses either 4 or 5 cases of the total of 29 were imputed for the intervention group (depending on outcome) and 3 or 4 of the 27 in the controls. Table 2, Table 3 show very similar results with and without multiple imputation (see also the later results section). When assessing sensitivity of the multiple imputations, results did not depend on the pattern of missingness. A conservative 10 multiple imputations for each analysis was used, given that no more than 10% of the data was missing. This is ample to account for variations between imputations (White, Royston, & Wood, 2011) and is further borne out by relative efficiencies of over 97% for all estimates of group differences associated with the multiple imputations.

**Table 2:** Comparisons of intervention and control group at one week and one month after ECS (intention-to-treat analyses).

	Intervention Mean ± SD^a^	Control Mean ± SD^a^	*t*	*d*	*SE*	*95% CI* for *d*	*p*
***One week after ECS***	*n* = 29	*n* = 27					
**Primary outcome:**							
Intrusive memories of ECS (diary) total	4.77 ± 10.71	9.22 ± 10.69	2.421*	0.647	0.280	0.106, 1.182	0.017
Acute Stress Disorder (1 week)							
ASDS Total score	20.64 ± 8.43	24.22 ± 8.40	1.558	0.417	0.275	−0.115, 0.945	0.120
ASDS re-experiencing subscale	5.69 ± 2.64	6.37 ± 2.64	1.879	0.503	0.277	−0.032, 1.033	0.060
ASDS avoidance subscale	2.14 ± 0.37	2.07 ± 0.37	0.793	0.212	0.273	−0.315, 0.737	0.428
ASDS arousal subscale	7.00 ± 3.33	8.37 ± 3.34	1.213	0.324	0.274	−0.205, 0.850	0.226
ASDS dissociation subscale	4.48 ± 1.86	4.74 ± 1.86	0.884	0.236	0.273	−0.291, 0.761	0.378
HADS Anxiety^b^ (1 week)	5.45 ± 3.39	6.34 ± 3.39	1.079	0.289	0.274	−0.239, 0.815	0.318
HADS Depression (1 week)	4.41 ± 3.79	4.12 ± 3.75	0.213	0.060	0.272	−0.465, 0.584	0.832
***One month after ECS***	*n* = 29	*n* = 27					
Posttraumatic stress disorder (1 month)							
PDS total score^c^	5.60 ± 7.61	6.93 ± 7.69	0.271	0.072	0.272	−0.453, 0.596	0.787
PDS hyperarousal subscale score	2.10 ± 3.18	2.43 ± 3.09	0.798	0.213	0.273	−0.314, 0.738	0.425
PDS reexperiencing subscale score	1.62 ± 2.39	2.10 ± 2.31	1.012	0.270	0.274	−0.258, 0.795	0.312
PDS avoidance subscale score	2.03 ± 3.63	2.48 ± 3.54	1.008	0.270	0.274	−0.258, 0.795	0.314
HADS Anxiety^b^ (1 month)	5.32 ± 3.79	5.85 ± 3.55	0.620	0.166	0.273	−0.360, 0.690	0.505
HADS Depression (1 month)^d^	4.35 ± 3.90	3.13 ± 4.04	−0.862	−0.231	0.273	−0.756, 0.296	0.389
	*n* (%)^a^	*n* (%)^a^	*χ*^*2*^(1)	Log (*OR*)	*SE*	*95%CI* for *OR*	*p*
PTSD diagnostic criteria (1 month)^1^	1.7 (5.9%)	7.3 (27.0)	2.87	1.884	1.113	0.730, 59.325	0.092
PDS re-experiencing cluster symptom count^2^	16.4 (56.6%)	17.0 (63.0%)	0.21	0.270	0.586	0.415, 4.138	0.645
PDS avoidance cluster symptom count^3^	3.0 (10.3%)	9.9 (36.7%)	3.90	1.646	0.834	1.003, 26.835	0.050
PDS hyperarousal cluster symptom count^4^	10.2 (35.2%)	13.6 (50.4%)	1.05	0.631	0.617	0.557, 6.344	0.307

*p < 0.05.

^a^ averages over 10 multiple imputations; ^b^*t*-test on untransformed data; ^c^*t*-test on log-transformed data; ^d^*t*-test on square root-transformed data; for all other outcomes a ranked *t*-test (non-parametric) was used.

^1^at least one re-experiencing, 3 avoidance and 2 hyperarousal symptoms on PDS, ^2^at least 1 re-experiencing symptom, ^3^at least 3 avoidance symptoms, ^4^at least 2 hyperarousal symptoms.

ECS = emergency caesarean section; ASDS = Acute Stress Disorder Scale; HADS = Hospital Anxiety and Depression Scale; PDS = Posttraumatic Diagnostic Scale; PTSD = Posttraumatic Stress Disorder.

**Table 3:** Comparisons of intervention and control group at one week and one month after ECS (per protocol analyses).

	Intervention Mean ± SD	Control Mean ± SD		*F*	*d*	*SE*	*95% CI* for *d*	*p*
***One week after ECS***	*n* = 25	*n* = 24						
**Primary outcome:**								
Intrusive memories of ECS (diary) total	3.54 ± 11.16	9.00 ± 9.32		10.94***	0.945	0.308	0.349, 1.532	0.002
	*n* = 24	*n* = 23						
Acute Stress Disorder (1 week)								
ASDS Total score	19.67 ± 6.03	24.48 ± 9.82		2.738	0.483	0.303	−0.100, 1.061	0.105
ASDS re-experiencing subscale	5.38 ± 1.74	7.43 ± 3.31		4.751*	0.636	0.306	0.046, 1.219	0.035
ASDS avoidance subscale	2.42 ± 1.02	2.83 ± 2.04		0.526	0.212	0.299	−0.364, 0.783	0.472
ASDS arousal subscale	8.00 ± 3.54	9.22 ± 3.98		1.369	0.341	0.300	−0.237, 0.915	0.248
ASDS dissociation subscale	3.87 ± 1.48	5.00 ± 2.30		1.692	0.380	0.301	−0.199, 0.955	0.200
HADS Anxiety^a^ (1 week)	5.21 ± 2.55	6.57 ± 3.98		*t* = 1.386	0.404	0.301	−0.176, 0.980	0.174
HADS Depression (1 week)	4.04 ± 3.28	4.00 ± 3.58		0.033	0.053	0.298	−0.519, 0.625	0.858
***One month after ECS***	*n* = 24	*n* = 23						
Posttraumatic stress disorder (1 month)								
PDS total score^b^	4.04 ± 4.41	7.39 ± 8.06		*t* = 1.112	0.324	0.300	−0.254, 0.898	0.272
PDS hyperarousal subscale score	1.46 ± 1.77	2.61 ± 2.97		1.424	0.348	0.301	−0.230, 0.922	0.239
PDS re-experiencing subscale score	1.25 ± 1.48	2.22 ± 2.75		0.968	0.287	0.300	−0.290, 0.860	0.330
PDS avoidance subscale score	1.33 ± 2.30	2.57 ± 3.55		2.011	0.414	0.302	−0.166, 0.990	0.163
	*n* = 23	*n* = 23						
HADS Anxiety^a^ (1 month)	5.17 ± 3.08	5.87 ± 3.81		*t* = 0.681	0.201	0.302	−0.380, 0.779	0.499
HADS Depression (1 month)^c^	4.26 ± 3.71	3.04 ± 3.39		*t* = 1.185	0.349	0.304	−0.235, 0.930	0.242
	*n* (%) (*n* = 24)	*n* (%) (*n* = 23)		*χ*^*2*^(1)	Log (*OR*)	*SE*	*95%CI* for *OR*	*p*
PTSD diagnostic criteria (1 month)^1^	1 (4.2%)	7 (30.4%)		4.268*	−2.309	1.118	0.011, 0.888	0.039
PDS re-experiencing cluster symptom count^4^ (N, %)	14 (58.3%)	15 (65.2%)		0.235	−0.292	0.603	0.229, 2.432	0.628
PDS avoidance cluster symptom count^2^ (N, %)	2 (8.3%)	9 (39.1%)		5.256*	−1.956	0.853	0.027, 0.753	0.022
PDS hyperarousal cluster symptom count^3^ (N, %)	8 (33.3%)	12 (52.2%)		1.683	−0.780	0.601	0.141, 1.490	0.195

*p < 0.05; ***p < 0.005.

^a^*t*-test on untransformed data; ^b^*t*-test on log-transformed data; ^c^*t*-test on square root-transformed data; for all other outcomes a ranked *t*-test (non-parametric) was used.

^1^at least one re-experiencing, 3 avoidance and 2 hyperarousal symptoms on PDS, ^2^ at least 3 avoidance symptoms, ^3^ at least 2 hyperarousal symptoms, ^4^ at least 1 re-experiencing symptom.

ECS = emergency caesarean section; ASDS = Acute Stress Disorder Scale; HADS = Hospital Anxiety and Depression Scale; PDS = Posttraumatic Diagnostic Scale.

Pairwise differences between variables were analysed using unpaired *t*-tests if the residuals obtained using these *t*-tests achieved normality with *p*-values above 0.05 using both the Kolmogorov-Smirnov and Shapiro-Wilk tests. Satterthwaite's correction was applied to the degrees of freedom of the *t*-test if the group variances were found to differ using Levene's test. If a pairwise comparison on an untransformed response produced residuals that did not achieve normality, then log, square root and reciprocal transformations were used and normality of the residuals re-assessed. If the use of transformations failed to induce normality in the residuals group differences were then analysed using ranked *t*-tests which are equivalent to Mann-Whitney tests (Conover & Iman, 1981).

To investigate the time course of intrusive traumatic memories reported in the daily diary over the first seven days after the ECS, a repeated measures analysis of covariance (ANCOVA) with time as a covariate was used. Frequency scattergraphs showing the distribution of the number of intrusive memories on each day for each group were plotted. A nonlinear time-series analysis was used to produce a nonparametric line of best fit, summarising the distribution of the number of intrusive traumatic memories on each day, smoothed from day to day over the seven-day period, by accounting for the number of intrusive traumatic memories at nearby time points (autocorrelation). This was achieved by fitting counts of the number of intrusive memories for each participant (*Y*) through time (*t*) with a generalized additive model (Hastie & Tibshirani, 1990):

(1) $$\begin{array}{l} Y\left(t\right)∼\text{Poisson}\left(u\left(t\;\right)\right)\\ \text{log}\left(\text{u}\left(\text{t}\;\right)\right)=\text{intercept}+\text{s}\left(\text{t,}\;4\right) \end{array}$$

where *u* is a random variable of time and *s* (*t*, 4) is the smoother with four effective degrees of freedom (as in (James et al., 2015)). Time series analyses were undertaken in R.

The content of the 475 intrusive traumatic memories written by participants in the diary entries were translated from French into English for readability by all the study team. Examples of intrusive memories include ‘*Seeing the fear on my husband's face when I was told I needed an emergency caesarean section’, ‘Seeing the face of the hospital doctor (surgeon)’, ‘I see myself reflected in the lights of the operating theatre’, ‘Seeing the eyes of a nurse in front of me in the operating theatre’*. Two researchers not involved in data collection and blind to group allocation independently checked all diaries for possible violations of diary instructions (i.e. where the participant had recorded an event that was unequivocally not an intrusive memory as per the instructions received e.g. unambiguously not a memory related to childbirth as verified with the participant). For example, one participant had noted on the diary that they had experienced breastfeeding problems, and confirmed with the researchers that they had not in fact experienced an intrusive memory on that occasion. This entry was therefore not counted. Agreement between the researchers was 100%. Out of 475 diary entries, 22 were identified as violations, thus 453 intrusive memories were used in the final analysis. 100% of raw data for the primary efficacy analysis of intrusive memories, and a randomly selected 10% of raw data for the secondary efficacy analyses, were checked for accuracy by a researcher not involved in data collection and blind to participant condition. No corrections were made.

## 2 Results

### 2.1 Baseline comparisons

There were no group differences regarding sociodemographic, obstetric and self-report measures (HADS and ASDS) at baseline (see Table 1). All participants were able to engage in the study procedures e.g. play the Tetris game via Nintendo while in the wake up room of the hospital after ECS (Fig. 5) and with the baby present, fitting in well around usual care in the hospital. However, three patients did not play Tetris for 10 min because they were each interrupted by important medical or care procedures.

### 2.2 Primary outcome

**Fig. 2:**
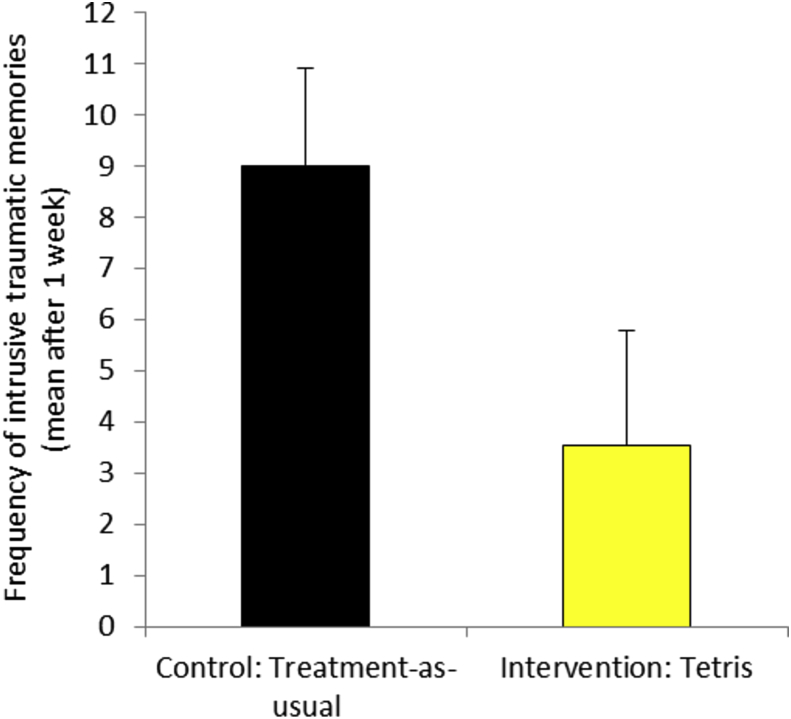
Mean number of intrusive traumatic memories recorded in the diary during the first 7 days following an emergency caesarean section for the Control (based on “per protocol” analysis): Usual care (*n* = 24; *M* = 9.00, *SD* = 9.32, *SEM* = 1.90) and Intervention: Tetris plus usual care (*n* = 25; *M* = 3.54, *SD* = 11.16, *SEM* = 2.23) groups, F = 10.94***, *d* = 0.945, *SE* = 0.308, 95% *CI* = 0.349, 1.532. ***p < 0.005. Error bars represent +1 *SEM*.

As predicted, intention-to-treat analyses showed that the intervention group had significantly fewer intrusive traumatic memories at 1 week than the control group (*M* = 4.77, *SD* = 10.71 vs. *M* = 9.22, *SD* = 10.69, *p* = 0.017, *d* = 0.647 [95% CI: 0.106, 1.182]. The “per protocol” analysis similarly showed that the intervention group had significantly fewer intrusive traumatic memories than the control group (*M* = 3.54, *SD* = 11.16 vs. *M* = 9.00, *SD* = 9.32, *p* = 0.002, *d* = 0.945 [95% CI: 0.349, 1.532]) over 1 week (Fig. 2).

### 2.3 Secondary outcomes

#### 2.3.1 Intention-to-treat analyses

Intention-to-treat analyses showed a trend of lower re-experiencing symptoms scores (ASDS subscale of acute traumatic stress disorder) at 1 week in the intervention group compared with the control group (*M* = 5.69, *SD* = 2.64 vs. *M* = 6.37, *SD* = 2.64, *p* = 0.060, *d* = 0.503 [95% CI: −0.032, 1.033]). There were no other significant group differences regarding the ASDS total score or other ASDS subscales, the HADS subscales, or the PDS total score or subscales (all *p* = ns; see Table 2, Table 3).

#### 2.3.2 “Per protocol” analyses

“Per protocol” analyses showed a significant group difference, with the intervention group having lower re-experiencing symptom scores (ASDS subscale of acute traumatic stress disorder) at 1 week than the control group (*M* = 5.38, *SD* = 1.74 vs. *M* = 7.43, *SD* = 3.31, *p* = 0.035, *d* = 0.636 [95% CI: 0.046, 1.219]) (Fig. 4). At 1 month, there were significant group differences regarding PTSD diagnostic criteria (intervention group: *n* = 1 (4.2%) vs. control group: *n* = 7 (30.4%), *p* = 0.039, Log (*OR*) = −2.309 [95% CI for *OR*: 0.011, 0.888]) and PDS avoidance symptom cluster count (intervention group: *n* = 2 (8.3%) vs. control group: *n* = 9 (39.1%), *p* = 0.022, Log (*OR*) = −1.956 [95% CI for *OR*: 0.027, 0.753]). No other significant group differences regarding the ASDS total score or other ASDS subscales, the HADS subscales, or the PDS total score or subscales were found (all *p* = ns; see Table 2, Table 3).

#### 2.3.3 Time course of intrusions

**Fig. 3:**
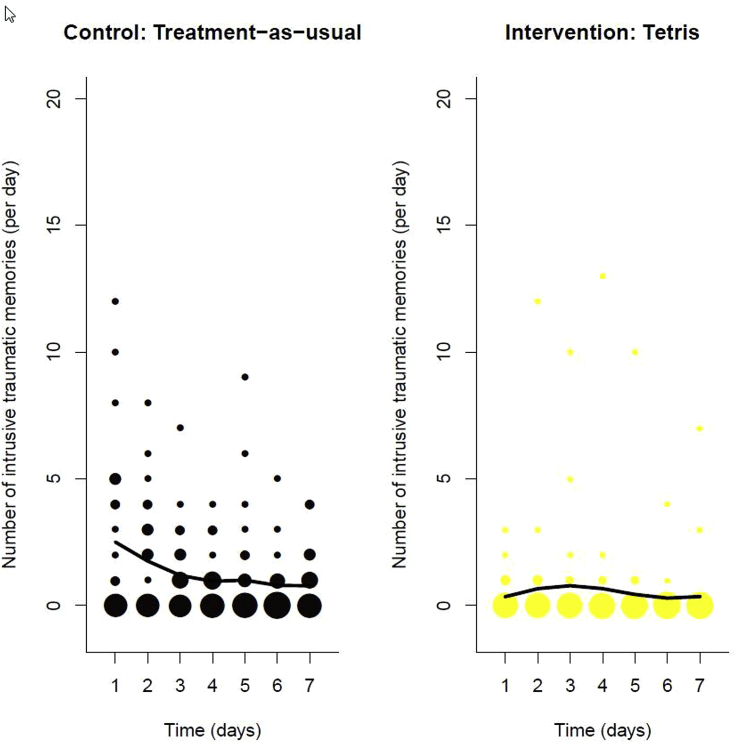
Frequency scattergraphs showing the time-course of the number of intrusive traumatic memories recorded in the diary from day 1 to day 7 for the Control: Usual care (*n* = 24) and Intervention: Tetris plus usual care (*n* = 25) groups. The size of the circles represents the number of participants who reported the indicated number of intrusive traumatic memories on that particular day, scaled separately for each condition. The solid lines are the fit of the generalized additive model (see Equation (1)) to summarise the number of intrusive traumatic memories through the seven-day period. Parametric analysis (repeated measures ANCOVA) confirms that there is a significant reduction in the number of intrusive traumatic memories through time (*F* = 6.412, *df* = 1,337, *p* = 0.012) and a significant difference between Control and Intervention groups (*F* = 10.5310, *df* = 1,337, *p* = 0.001) on the overall number of intrusive traumatic memories.

The non-linear time-series analysis to investigate the time course of intrusive traumatic memories over these first seven days showed that the number of intrusive traumatic memories remained very low across the seven days in the intervention group, whereas in the control group the number was higher across the seven days, particularly in the first two days (Fig. 3). Results of the repeated measures ANCOVA confirmed a significant reduction in the number of intrusive traumatic memories through time (*F* = 6.412, *df* = 1,337, *p* = 0.012), and critically, in line with hypotheses, a significant difference between control and intervention groups (*F* = 10.5310, *df* = 1,337, *p* = 0.001) on the overall number of intrusive traumatic memories, with fewer in the intervention group.

**Fig. 4:**
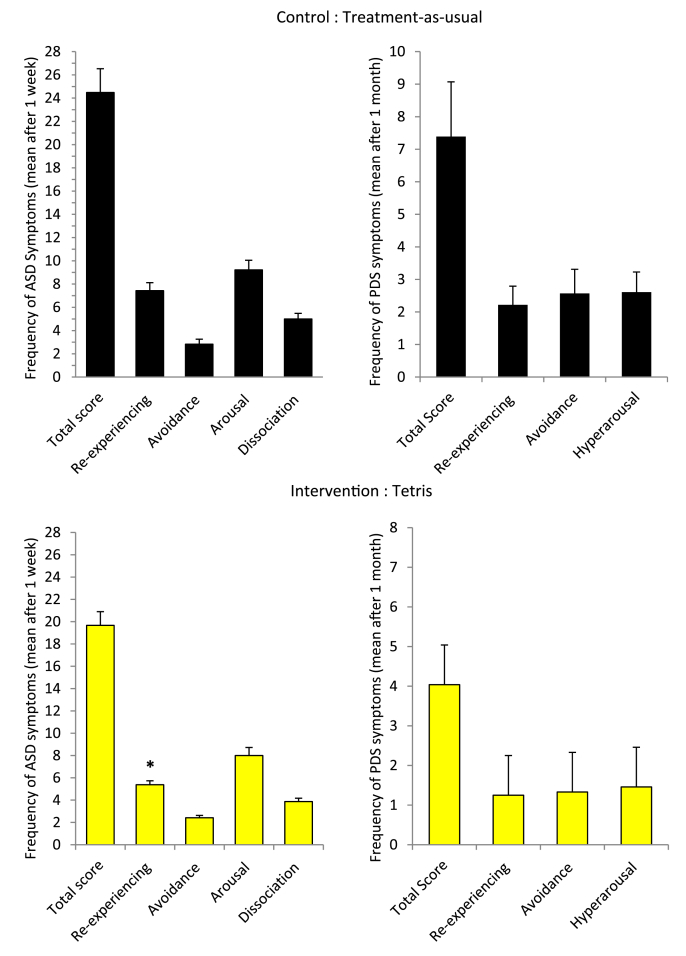
Mean number of self-reported acute stress disorder (ASD) symptoms after 1 week and posttraumatic stress disorder (PTSD) symptoms after 1 month following an emergency caesarean section (based on “per protocol” analysis) for the Control group: Treatment-as-usual and Intervention group: Tetris. Asterisks indicate a significant difference between groups (*p < 0.05). Error bars represent +1 *SEM*.

**Fig. 5:**
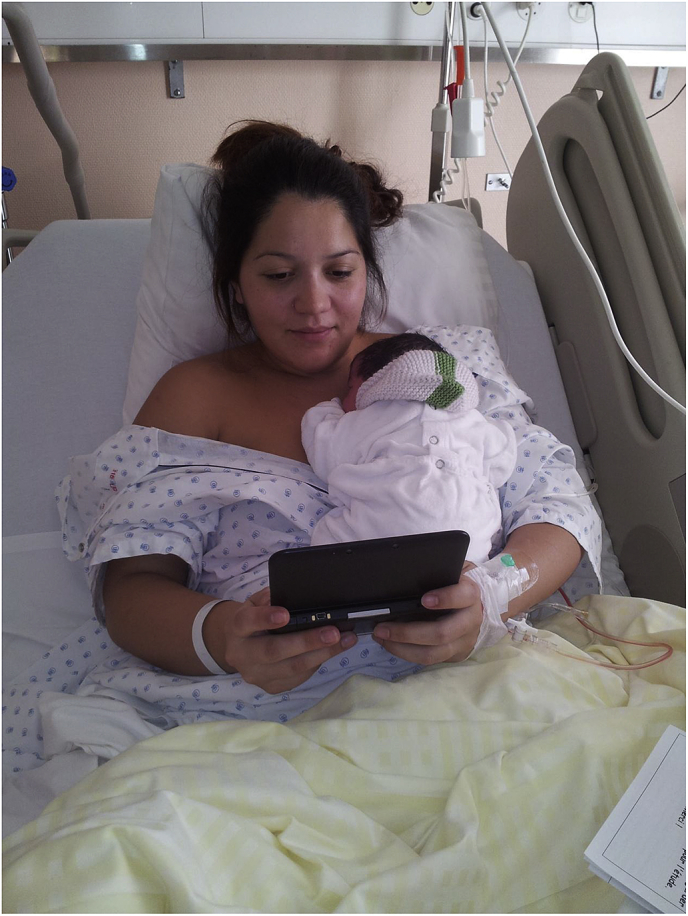
Horsch, A. (Photographer). (2013). Study participant carrying out the cognitive task procedure via Nintendo DS [photograph]. Department Woman-Mother-Child, University Hospital Lausanne, Switzerland. With permission.

#### 2.3.4 Participant feedback and experience of the intervention

The majority of participants found the Tetris intervention acceptable, with 72% rating it as *rather* to *extremely acceptable* (12% *not at all acceptable*, 16% *slightly acceptable*, 40% *rather acceptable*, 20% *very acceptable*, 12% *extremely acceptable*). When asked if they found it useful to play Tetris, 31.8% answered *not at all useful*, 27.3% *slightly useful*, 36.4% *rather useful*, and 4.5% *extremely useful*. In response to the question as to whether they would recommend the Tetris intervention to someone else, 54.5% answered ‘*yes*’ and 45.5% ‘*no*’.

No participant reported any harmful effects and no serious incident was reported over the course of the study.

## 3 Discussion

This study is the first to our knowledge to demonstrate the successful prevention of posttraumatic stress reactions (intrusive memories of trauma) in mothers post-ECS - and does so by a relatively simple cognitive intervention. Crucially, our intention-to-treat analysis provides evidence that the frequency of intrusive traumatic memories after a traumatic childbirth (primary outcome) is reduced by 48% after engaging in the brief cognitive task procedure (including playing the computer-game Tetris via Nintendo DS) for c.15 min during the first 6 h following ECS and while still in the hospital environment. The time course of intrusive traumatic memories reported in the daily diary over the first seven days after the ECS differs significantly between the intervention and control group, particularly in the first two days. This is critically the time during which the first contact with the baby and breastfeeding are established. Our “per protocol” analyses showed that mothers in the intervention group also reported reduced acute stress disorder symptoms (re-experiencing) after 1 week. This safe and feasible technological intervention was perceived to be acceptable by women who had just given birth, and was played with ease while in bed with their newborn child (see Fig. 5). Results demonstrate the successful early application of contemporary cognitive science to develop an evidence-based clinical intervention in a traumatised postnatal population, harnessing relatively simple and readily available technology.

The size of reduction observed in the number of intrusive memories of trauma (i.e. by 48%) is comparable with that found in previous laboratory-based experiments showing that Tetris can significantly reduce the frequency of intrusive memories following exposure to traumatic film material (e.g., 58% (Holmes et al., 2009); Our findings thus demonstrate a promising clinical translation from the laboratory to the real world – here the postnatal ward.

The study was powered for our primary outcome (the number of intrusive traumatic memories at one week post-trauma, i.e. within this acute period specifically), and significant between-group differences were not found for our secondary outcomes using intention-to-treat analyses, including PTSD symptoms/diagnosis at one month. Thus, it cannot be concluded from this study whether or not there are longer term effects in terms of PTSD prevention. However, if small effect sizes for symptom outcomes at one month (e.g., *d* = 0.271 for PDS total score, with 5.9% in the intervention group vs. 27% in the control group meeting diagnostic criteria for PTSD), as in the current study, were also to be found in a subsequent larger trial powered for these outcomes, this would be highly promising for patient care given the brief and inexpensive nature of the intervention.

Using “per protocol” analyses, we found a significant group difference regarding the mean number of self-reported acute stress disorder (ASD) symptoms after 1 week in addition to the significant group difference of number of intrusive traumatic memories at 1 week post-trauma. The convergence of the intention-to-treat and “per protocol” analyses, and between both diary-recorded and questionnaire-measured intrusions (the ASDS re-experiencing subscale) supports the robustness of our primary analysis, and suggests that a larger trial, powered to detect effects on PTSD symptoms/diagnosis, is warranted, allowing the implications for PTSD prevention to be explored.

While the results of this study would need replication in a large randomized controlled trial before firm recommendations for clinical practice could be made, the results are particularly promising given the nature of the intervention tested: the technological intervention is feasible within the context of a busy maternity hospital; it does not take up much time and does not interfere with important nursing interventions that take place following an ECS. It meets various requirements of a universal intervention (Gordon, 1983), as it easily accessible, easy to administer without the use of a specialist therapist and cost-effective. Furthermore, it does not rely on using a particular language, appears harmless, and is acceptable to patients. Future research for development as is warranted given its potential scalability to help mothers and their offspring internationally. This intervention approach could also be applied in other traumatised populations than ECS, such as other types of patients who have experienced trauma in hospital settings (e.g. after cardiac surgery), and thus have significant wider public health interest. Indeed, since we also lack preventative treatments for PTSD in the general population (Roberts, Kitchiner, Kenardy, & Bisson, 2009), ECS offers trauma researchers the opportunity of a unique test platform - the traumatic event (ECS) is relatively standardised, as is the hospital setting in which a new intervention can be tested.

Our study also has important implications for research on the immediate response to a traumatic event, such as ECS. As previously stated, this is the first early intervention delivered in the hours following traumatic childbirth (either pharmacological or psychological) that has been shown to be effective at reducing acute posttraumatic stress reactions (the number of intrusive memories) in postnatal women at least over the first week – a very important week for maternal attachment and caring of the newborn infant. Historically there has been a pause in early intervention research after trauma because of evidence that interventions such as “psychological debriefing” could interfere with natural recovery following exposure to other types of traumatic events, such as road traffic accidents (Rose, Bisson, Churchill, & Wessely, 2009), and were ineffective following traumatic childbirth (Bastos et al., 2015). Engaging in a cognitive, visuospatial task procedure as we have deployed here is notably different from “psychological debriefing” therapy: it is based on the hypothesis and experimental data that tasks such as Tetris reduce the occurrence of subsequent intrusive memories of the trauma by competing with sensory aspects of the trauma memory before it has been fully consolidated, thereby reducing the frequency of recurrence of sensory intrusive memories of the trauma (Holmes et al., 2009, Holmes et al., 2010). Further research is needed to continue to test underlying mechanisms and on the optimisation of the intervention. For example, it would be of interest to increase the number of doses or duration of Tetris game play beyond 15 min. Varying the time allows may also permit future mechanistic analyses testing whether duration or quality of game play relates to number of intrusions (see also (James et al., 2015)). Future studies should also seek to monitor sleep since this also have a role in intrusive memory formation (Porcheret, Holmes, Goodwin, Foster, & Wulff, 2015).

Further limitations of this study include the use of usual care control group rather than an active control. Within the current design, it is not possible to separate out potential effects of expectancy (of researchers or participants) from the hypothesised specific effects of the intervention administered. Future research should seek to test whether there is a specific effect of adding an engaging visuospatial cognitive task (here Tetris, but also others) as per our hypothesis or a general effect of adding in any task. However, the choice of an active control group is not straightforward, as there is no standard treatment to use as a comparator, while there are potential ethical implications of some alternative procedures with this vulnerable group, and some other tasks are also likely to be beneficial. For example, an alternative to a visual computer game would be a verbal computer game, but in some studies such games have been found to *increase* the number of intrusive traumatic memories indicating possible harmful effects (Holmes et al., 2009). Other preventive psychological treatment approaches, such as “psychological debriefing”, are counter-indicated (Rose et al., 2009). Furthermore, we do not predict that results would be restricted to Tetris alone – rather any engaging visuospatial task such as other visual computer games (e.g. Candy Crush or drawing) – would also be expected to have effects (James, Visser, Landkroon, & Holmes, 2017). Ideally, a bespoke form of active ‘attention placebo’ could be developed in future studies.

The aim of the current proof-of-principle study was not to try to separate out such effects at this stage, but rather to provide a first investigation of potential feasibility, acceptability, and efficacy (over and above usual care). This is a critical first step and important in this new patient group - women who had just in the last hours experienced the near-death of themselves or their infant – before dismantling effects. It is interesting that the results found are highly specific, and the effect sizes reduce as one moves away from the measures most closely linked to the hypothesised active mechanism targeted by Tetris (i.e. from the diary-recorded intrusions, to re-experiencing symptoms, to hyperarousal, and so on). We might expect demand/expectancy-driven effects to show more broad across-the-board benefits rather than the specific pattern observed here.

The analysis conducted mid-way might have led to a potential positive expectancy bias. The reliance on self-report data for both primary and secondary outcomes could be augmented in future trials and full blinding of researchers implemented. In terms of intervention uptake, a high proportion of mothers who were approached agreed to take part (69.1%). This is notable given the nature of traumatic childbirth, and also comparable studies in hospital emergency departments (10% for prolonged exposure trial (Rothbaum et al., 2012); or 8% for pharmacotherapy trial (Stein, Kerridge, Dimsdale, & Hoyt, 2007)). Future studies should add a measure to more fully assess the acceptability of the intervention to those who chose *not* to take part in the study (see Fig. 1: No interest in research study, n = 5; Does not like computer games, n = 2: total % of participants approached = 8.64%). In terms of adherence, while 89.7% adhered to the game play component, it is noted that of those who did consent to take part, 3/29 played Tetris for less than the minimum period of 10 min and this should also be explored. In terms of patient satisfaction, participant feedback and experience of the intervention ratings also indicate that there are useful aspects of acceptability to explore in future. In addition, future studies should add clinician-administered diagnostic interviews to assess psychopathological symptoms. The study was initially conceived as an extension of previous laboratory research into a clinical population and was not registered prospectively as a clinical trial. However, by randomizing participants to complete a task and measuring subsequent health-related outcomes it is now considered de facto a clinical trial and future trials should be prospectively registered. These above limitations reflect the early-phase nature of the study, the results of which now justify carrying out larger-scale trials with sufficient resources to allow e.g. blinding of assessors. In addition, the majority of our sample had a partner and had a university education and the results may therefore not automatically be generalized to single women or women with different educational backgrounds.

It will be important to test replication and extension of these findings in a larger RCT with a longer follow-up period and to confirm specific mechanisms of change, as well as impact on not only the mother but also the child. Future research is needed to determine what the optimal time window and duration for this type of intervention. Extension of the intervention approach should be examined for those patients who would need to undertake it at longer time intervals post-trauma by drawing on reconsolidation update mechanisms (James et al., 2015). There is recent evidence that the intervention approach is also effective in reducing intrusions in patients presenting to the Emergency department comprising a conceptual test of replication of current findings (Iyadurai et al., 2017). Further research in different clinical samples and settings is warranted.

In summary, this translational proof-of-concept study shows that the number of intrusive traumatic memories and acute stress symptoms after traumatic childbirth can be reduced, using a cognitive computerized intervention within 6 h of a traumatic childbirth - an ECS. It represents a first step in the development of a hypothesis-driven early intervention to prevent postnatal posttraumatic stress symptoms (intrusive memories of trauma) – something which would be of great potential benefit to both mother and child.

## Funding

This research did not receive any specific grant from funding agencies in the public, commercial, or not-for-profit sectors.

## Declaration of conflicting interests

The authors declared that they had no conflicts of interest with respect to their authorship or the publication of this article.
